# A Functional Polymorphism in the Promoter Region of MicroRNA-146a Is Associated with the Risk of Alzheimer Disease and the Rate of Cognitive Decline in Patients

**DOI:** 10.1371/journal.pone.0089019

**Published:** 2014-02-25

**Authors:** Lili Cui, You Li, Guoda Ma, Yan Wang, Yujie Cai, Shengyuan Liu, Yanyan Chen, Jia Li, Yuliu Xie, Gen Liu, Bin Zhao, Keshen Li

**Affiliations:** 1 Guangdong Key Laboratory of Age-Related Cardiac and Cerebral Diseases, Affiliated Hospital of Guangdong Medical College, Zhanjiang, PR China; 2 Clinical Research Center of Guangdong Medical College, Affiliated Hospital of Guangdong Medical College, Zhanjiang, PR China; 3 Department of Chronic Disease, Shenzhen Nanshan Center for Chronic Disease Control, Shenzhen, PR China; 4 Intensive Care Unit, Affiliated Hospital of Guangdong Medical College, Zhanjiang, PR China; 5 Institute of Neurology, Affiliated Hospital of Guangdong Medical College, Zhanjiang, PR China; Duke-NUS Graduate Medical School, Singapore

## Abstract

miR146a is well known for its regulatory role in the immune response and inflammation. Recent studies have demonstrated the links between miR146a and Alzheimer disease (AD) and suggested that miR146a may be involved in neuroinflammation and the metabolism of amyloid-β (Aβ), which are critical events in AD pathology. Although genetic studies have focused on the association between the miR146a gene and susceptibility to several diseases, no association study of miR146a variability with AD has been conducted. In this report, we performed a case-control association study to analyze the genotype and allele distributions of the miR146a, rs2910464 and rs57095329 polymorphisms in a Chinese population consisting of 292 AD cases and 300 healthy controls. We found a significant difference in the genotypes and allele frequencies of rs57095329 between the AD cases and the controls (p = 0.0147 and p = 0.0184, respectively), where the AA genotype of rs57095329 was associated with an increased risk of AD as well the cognitive decline in AD patients. Additionally, the AA genotype of rs57095329 exhibited significantly higher miR146a expression than the GG+GA genotypes of rs2910164 in the peripheral blood cells (PBMCs) of healthy individuals and had a stronger effect on the production of IL-6 and IL-1β when the cells were stimulated with LPS. Our data provide preliminary evidence that the rs57095329 polymorphism in the miR146a promoter is involved in the genetic susceptibility to AD, and this risk AA genotype may increase the expression of miR146a and influence certain proinflammatory cytokines, thus playing a role in the pathogenesis of AD.

## Introduction

Alzheimer disease (AD) is a common age-related neurodegenerative disease that is characterized by the progressive loss of memory and cognitive decline. The mechanisms of AD occurrence are complex; currently, the aggregation of amyloid-β (Aβ) peptide in the brain is considered to be the initiating factor, whereas the immunity and inflammatory responses that are caused by the deposition of Aβ in the brain also play fundamental roles in the pathogenesis of AD [1]. Mutations in three genes, amyloid precursor (APP), presenilin 1 (PSEN1) and presenilin 2 (PSEN2), play extremely important roles in the metabolism of Aβ, and these mutations have been identified as risk factors for Early Onset Alzheimer Disease (EOAD) [2]. Currently, only the ε4 allele of apolipoprotein E (ApoE) is considered a risk factor for Late Onset Alzheimer Disease (LOAD) [3].

MicroRNAs (miRNAs) are small noncoding RNAs that are 20–23 nucleotides in length and regulate gene expression in a sequence-specific manner [4]. Many miRNAs are abundant in the nervous system [5] and are involved in various biological and pathological processes in the brain [6]. One of the conserved and nervous system-specific miRNAs is miR146a, which is well known for its important regulate role in the immune response and inflammation [7], [8]. Recently, there has been increasing evidence to support the hypothesis that miR146a operates as an important epigenetic regulator in various pathways involved in AD pathogenesis. The aberrant expression of miR146a has been observed in different AD transgenic mouse models and human AD brains [9], [10], and this upregulation of miR146a was detected in anatomical regions exhibiting AD neuropathology while it was unchanged in regions of the brain that were unaffected [11]. In addition, it is worth noting that miR146a is an NFkB target gene and can directly target CFH and IRAK1 mRNA and ultimately play a role in increasing the inflammation response in the brains of AD patients [12], [13], [14].

miR146a is located on chromosome 5q33.3. Previous studies confirmed that several single-nucleotide polymorphisms (SNPs) of miR146a have functional importance and can modify the expression level of mature miR146a [15], [16]. The SNP rs2910146 (G/C) is located in the stem region, opposite of the mature miR146a sequence (C allele) and results in a mismatch in the stem structure of pre-miR146a, leading to a decrease in the total amount of mature miR146a, which in turn influences the transcription of target genes and the pathogenesis of the disease [16]. Several immune and inflammatory-related diseases are associated with rs2910164 polymorphisms, such as papillary thyroid carcinoma [16]. Behçet's disease [17], ulcerative colitis [18], adult glioma [19] and prostate cancer [20] Recently, another genetic variant, rs57095329 (A/G), in the miR146a promoter was reported to affect the level of mature miR146a by reducing the protein-binding affinity of the miR146a promoter, and this polymorphism has been associated with systemic lupus erythematosus (SLE) susceptibility [15]. However, to the best of our knowledge, no studies have examined the association of miR146a polymorphisms with the risk of AD. In the present study, we conducted an association analysis to ascertain whether the two functional polymorphisms of miR146a could contribute to the risk of AD in the Chinese population.

## Materials and Methods

### 2.1 Ethics statement

All experiments on human subjects were conducted in accordance with the Declaration of Helsinki, written informed consent was obtained from all the enrolled participants and this study was approved by the Ethics Committee of the Affiliated Hospital of Guangdong Medical College.

### 2.2 Patient and control samples

In total, 292 AD patients and 300 controls were included in this case–control study. All AD subjects were enrolled at the Department of Chronic Disease, Shenzhen Nanshan Center for Chronic Disease Control between April 2008 and Febuary 2013. A clinical diagnosis of probable AD was established according to the criteria of the National Institute of Neurological and Communicative Disorders and Stroke and the Alzheimer disease and Related Disorders Association (NINCDS–ADRDA). The Mini-Mental State Examination (MMSE) was primarily used to evaluate cognitive impairment [21]. Patients with evidence of vascular and “mixed” dementia were excluded. The control groups were confirmed to be healthy and neurologically normal according to their medical history, general examinations, laboratory examinations and MMSE scores (score>28). All subjects who had a history of cancers, coronary artery disease, cerebrovascular diseases, diabetes and any neurological disorders were excluded. The demographic data and clinical features of the patients included as AD and healthy control are shown in Table 1. Moreover, 196 AD patients from the AD group were followed up for two years to monitor the disease progression. The MMSE scores were recorded in each patient and the dropped MMSE scores was calculated during the two-year follow-up.

**Table 1:** Demographic data and clinical features of patients with AD Patients and healthy controls.

	Gender (male/female)	Ages (years) (means±SD)	MMSE scores	ApoEε4 carrier (no.)
**AD(292)**	121/171	74.1±7.72	18.4±6.22	121
**Controls (300)**	130/170	73.6±8.29	28.7±0.82	54
**p-value**	0.6201	0.4479	<0.001	<0.0001

SD: standard deviation; no: number.

### 2.3 Genotyping

Genomic DNA was isolated from peripheral blood samples using blood Genomic DNA Extraction Kit (Tiangen, China). The DNA was genotyped using the ABI PRISM SNapShot method (Applied Biosystems, Foster, CA). The PCR primers used for polymorphism site rs2910164 were: 5-GAACTGAATTCCATGGGTTG-3 and 5-CACGATGACAGAGATATCCC-3. The primers used for rs57095329 were 5-TCATTGGGCAGCCGATAAAG-3 and 5-AGGAAGTTCTGGTCAGGCG-3. Briefly, the SNapShot reaction was carried out in a 10 µl final volume containing SNapShot Multiplex Ready Mix (5 µl), primer mix (0.02–0.6 µmol/L), and templates (4 µl) consisting of the multiplex polymerase chain reaction (PCR) products, which had been purified with the QIAquick PCR Purification Kit (QIAGEN, Hilden, Germany). The cycling program included 25 cycles of 94°C for 30 seconds, 57°C for 30 seconds, and 72°C for 40 seconds. Extension products were purified by a 15-minute incubation with 1U of shrimp alkaline phosphatase (Promega, Madison, WI) at 37°C and a subsequent 15-minute incubation at 80°C to inactivate the enzyme. The purified products (0.5 µl) were mixed with 9 µl of formamide and 0.5 µl of GeneScan-120 LIZ Size Standard (Applied Biosystems) and separated by capillary electrophoresis (ABI PRISM310 Genetic Analyzer; Applied Biosystems). The results were analyzed with GeneMapper 3.0 software (Applied Biosystems). For quality control, random duplicate samples (5%) were run for each sequence analysis.

### 2.4 Mononuclear cells isolation and Elisa

Peripheral blood mononuclear cells (PBMCs) were isolated using density gradient centrifugation method with Lymphoprep™ (Axis-Shield PoCAS, Oslo, Norway). In brief, blood samples were mixed with equal volume of 0.9% NaCl. The diluted blood was then slowly added to tubes containing a Ficoll premium solution to make the blood layered upon the Ficoll. Samples were centrifuged at 800×g for 30 min at room temperature. After centrifugation, the mononuclear cells form a distinct band at the medium interface. The cells were then shifted to other tubes using Pasteur pipette without removing the upper layer and washed with 0.9% NaCl. Then samples were centrifuged again at 250×g for 10 min. The mononuclear cells were harvested and stored at −80°C. Isolated PBMCs cells (2×10^6^ cells per well) were seeded in 24-well plates and cultured in RPMI medium 1640 supplemented with 10% fetal calf serum (FCS), 100 U/ml penicillin and 100 µg/ml streptomycin. For the measurement of IL-1β and IL-6, the PMBCs were incubated with 50 ug/L lipopolysaccharide (LPS) (Sigma, USA) for 24 h. The concentrations of IL-1β and IL-6 in the supernatants of PBMCs were detected using the human Elisa kit for IL-6 and IL-1β (R&D Systems, Minneapolis, MN, USA) according to the manufacturer's instructions.

### 2.5 RNA extraction and real-time PCR

For the subjects with the GG genotype of 2910164 and the GG geneotype of rs57095329 in patients were relatively less, we choose the samples with the two genotypes in healthy subjects for priority in order to meet the statistical requirements, while other individuals were selected at random for the healthy subjects. In total 47 healthy **i**ndividuals were enrolled for the RNA extraction, one sample with the AA genotype of rs57095329 was failed to detect Relative-expression of miR146a, so 47 samples for the known genotype of rs2910164 and 46 samples for the known genotypes of rs57095329 were finally used for the following experiments. Total RNA was extracted from PBMCs using Trizol, followed by reverse transcription using transcriptase kit (Invitrogen, USA), The quantity of mature miR146a was determined by Taqman MicroRNA Assay kit (Applied Biosystems, USA), The assay were performed on a 7500 real time instrument (Applied Biosystems, USA). Data analysis was performed with the software provided by the manufacturer, using the 2^△DDCt^ method to determine the relative level of miR-146a and expressed as a fold-difference to the relevant control. Values were normalized to snRNA U6.

### 2.6 Statistical analyses

All analyses were performed using SPSS (version 19.0) software. Genotype and allele frequencies were estimated by counting. Hardy–Weinberg equilibrium between expected and observed genotype distributions were assessed using the Chi-square test. Allele and genotype distributions were compared using the Chi-squared test. Genotypic distributions were performed using the Student's t-test and the Chi-square test when appropriate. Analyses were repeated in subgroups stratified by age and ApoEε4 carrier status. Association was expressed as odds ratios (OR) or risk estimates with 95% confidence intervals (CI). Haplotype analyses were conducted using Haploview software (version 3.2.0). The non-parametric Mann Whitney test was used to compare the miR146a expression and the production of IL-6 and IL-1β among three genotypes of rs2910164 and rs57095329, respectively. The data are presented as percentage frequencies or means±SD. Two-Tailed P values<0.05 was considered to be statistically significant.

## Results

### 3.1 Demographics

A total of 292 patients diagnosed with AD and 300 unrelated gender and age-matched controls were analyzed in our study. The demographic and clinical characteristics of the study subjects are summarized in Table 1. As shown, the mean age was 73.6 years (±8.3 years) for the AD patients and 74.1 years (±7.7 years) for the control subjects; the gender (male-to-female) ratio was 1∶1.41 in the case group and 1∶1.31 in the control group. The distributions of the age and gender did not significantly differ between the AD patients and controls (P = 0.6201 and 0.4479, respectively). The MMSE score was significantly lower in the AD patients than in the controls (p<0.001). Moreover, the ApoEε4 allele frequencies were significantly elevated in the AD patients than in the controls, as expected (p<0.0001).

### 3.2 The association of miR146a polymorphisms between AD cases and controls

All of the enrolled samples were successfully genotyped for the rs2910164 and rs57095329 SNPs, and the association between the genotype and the risk of AD was analyzed using the chi-squared test. All genotype distributions were in Hardy-Weinberg equilibrium in the AD cases and controls. The allele and genotype frequencies of the miR146a polymorphisms in the entire study population are shown in Table 2. Neither the genotype nor the allele in rs2910164 showed significant differences between the AD cases and the controls (p = 0.5901 and 0.3580, respectively), and no significant difference was observed in the subgroups with the ApoEε4 mutation (ApoEε4 (+): genotypes p = 0.8863 and alleles p = 0.7347, ApoEε4 (−): genotypes p = 0.4033 and alleles p = 0.2364) or the age of disease onset subgroups (LOAD: genotypes p = 0.8416 and alleles p = 0.6605, EOAD: genotypes p = 0.5957 and alleles p = 0.3226) (Table 3). There were significant differences in the genotype and allele frequencies of the rs57095329 SNP between the AD cases and controls (genotypes P = 0.0147 and alleles P = 0.0184). In a recessive model (AA vs. GA + GG) a significant difference was observed in the AD cases compared with the controls (P = 0.0037), indicating that the rs57095329 polymorphism should be a risk factor for AD (Table 2).When these data were stratified for the presence or absence of the ApoEε4 mutation, the allele and genotype distributions of rs57095329 between the AD patients and controls remained significantly different in both the ApoEε4 (+) (genotypes P = 0.0471 and alleles P = 0.0459) and ApoEε4 (−) subgroups (genotypes P = 0.0283 and alleles P = 0.0462) (Table 3). Moreover, in the EOAD subgroup, the genotype and allele distributions were significantly different between the cases and controls for the rs57095329 SNP (genotypes p = 0.027 and alleles p = 0.0042) (Table 3). However, no significant differences in genotype or allele frequencies were observed between the cases and controls in the LOAD subgroup (genotypes p = 0.5957 and alleles p = 0.3226). We further performed a haplotype-based association analysis of rs2910164 and rs57095329. However, no significant associations were observed between these haplotypes and AD (Table S1).

**Table 2:** Frequencies of miR146a genotypes and alleles in AD cases and controls.

	AD patients n (%)	Controls n (%)	OR (95% CI)	P-value
**rs2910164 G>C**				
** GG**	32(10.96)	36(12.00)		0.5901
** GC**	140(47.95)	153(51.00)		
** CC**	120(41.09)	111(37.00)		
** GC+CC**	260(89.04)	264(88.00)	1.108 (0.67–1.80)	0.6912
** GC+GG**	172(58.90)	189(63.00)	0.8418 (0.61–1.17)	0.3071
** G alleles**	204(34.93)	225(37.50)		
** C alleles**	380(65.07)	375(62.50)	1.118 (0.88–1.40)	0.3580
**rs57095326 G>A**				
** GG**	6(2.05)	9(3.00)		0.0147
** GA**	54(18.49)	84(28.00)		
** AA**	232(79.45)	207(69.0)		
** GA+AA**	286(97.95)	291(96.00%)	1.474 (0.52–4.20)	0.4644
** GA+GG**	60(20.54)	93(31.00)	0.5756 (0.40–0.84)	0.0037
** G alleles**	66(11.30)	102(17.00)		
** A alleles**	518(88.70)	498(83.00)	1.592(1.14–2.22)	0.0060

**Table 3:** Distribution of the rs2910164, rs57095329 genotypes and alleles among subgroups in case and controls.

rs2910164	Genotype n (%)				Allele n (%)			
Characters(n)	CC	GC	GG	p-value	C	G	OR (95% CI)	p-value
**ApoEε4(+)**								
AD (121)	49(40.50)	53(43.80)	19(15.70)	0.8863	151(62.39)	91(37.60)	1.082(0.69–1.70)	0.7347
Control (57)	21(36.84)	27(47.37)	9(15.79)		69(60.53)	45(39.47)		
**ApoEε4(−)**								
AD (171)	71(41.52)	87(50.87)	13(7.60)	0.4033	229(66.96)	113(33.04)	1.192(0.89–1.60)	0.2364
Control (243)	90(37.03)	126(51.85)	27(11.11)		306(62.96)	180(37.03)		
**LOAD(>65 years)**								
AD(189)	70(37.03)	95(50.26)	24(12.70)	0.8416	235(62.17)	143(37.83)	1.067(0.80–1.40)	0.6605
Control(193)	66(34.19)	102(52.85)	25(12.95)		234(60.62)	152(39.38)		
**EOAD (≤65 years)**								
AD(103)	50(48.54)	45(43.69)	8(7.77)	0.5957	145(70.39)	61(29.61)	1.231(0.82–1.86)	0.3226
Control(107)	45(42.06)	51(47.66)	11(10.28)		141(65.89)	73(34.11)		

rs57095329 (n)	Genotype n (%)				Allele n (%)			
Characters	AA	GA	GG	p-value	G	A	OR (95% CI)	p-value
**ApoEε4(+)**								
AD (121)	95(78.51)	26(21.49)	0(0.00)	0.0471	26(10.74)	216(89.26)	0.5331(0.29–0.99)	0.0459
Control (57)	38(66.67)	17(29.82)	2(3.51)		21(18.42)	93(81.58)		
**ApoEε4(−)**								
AD (171)	137(80.12)	28(16.37)	6(3.51)	0.0283	40(11.70)	302(88.30)	0.6623(0.44–0.99)	0.0462
Control (243)	169(69.55)	67(27.57)	7(2.88)		81(16.67)	405(83.33)		
**LOAD(>65 years)**								
AD(189)	147(77.78)	38(20.11)	4(2.11)	0.0920	46(12.17)	332(87.83)	0.7528(0.50–1.14)	0.1773
Control(193)	135(69.95)	56(29.02)	2(1.03)		60(15.54)	326(84.45)		
**EOAD (≤65 years)**								
AD(103)	85(82.52)	16(15.53)	2(1.94)	0.0270	20(9.71)	186(90.29)	0.4403(0.25–0.78)	0.0042
Control(107)	72(67.29)	28(26.17)	7(6.54)		42(19.63)	172(80.37)		

### 3.3 The association between miR146a polymorphisms and cognitive decline

**Figure 1:**
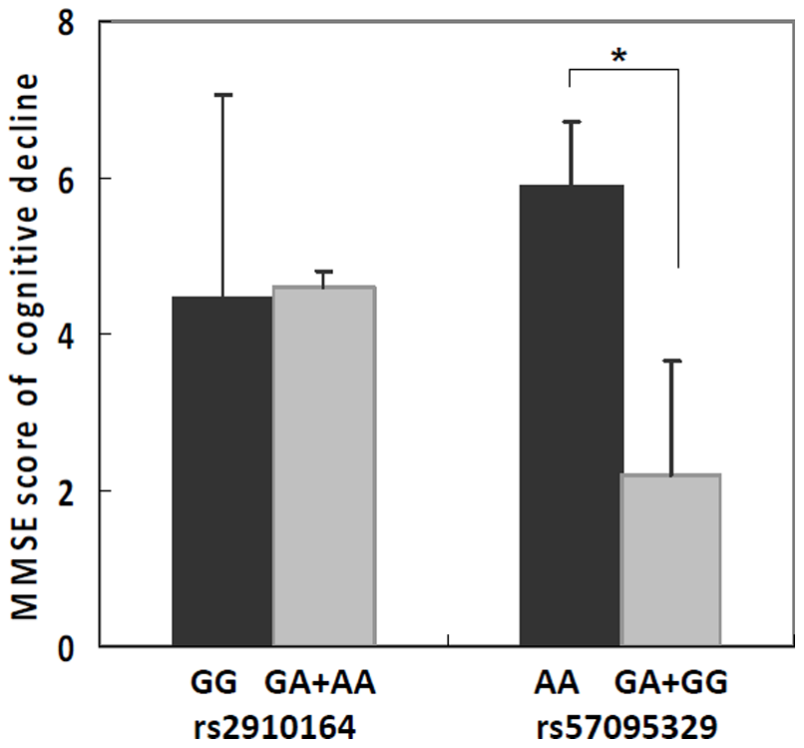
Genotype distribution of the miR146a polymorphisms in AD patients stratified according to the decreased MMSE scores of cognitive decline in AD patiemts (n = 196) with two-year periods. The decreased MMSE scores derived from SNP rs2910164 for GG (n = 23) and GC+CC (n = 173) genotypes and SNP rs57095329 for AA (n = 125) and GG+ GA (n = 71) Genotypes. Values are presented as the means±SD (*p<0.05).

The genotype distribution of miR146a was further analyzed in a subset of AD patients who were clinically diagnosed with AD and were followed up for 2 years to assess their cognitive decline. The decreased MMSE scores in 196 AD patients were recorded and then stratified by their genotypes of miR146a polymorphisms. As shown in Figure 1, The SNP rs2910164 showed no significant association with the cognitive deterioration of the AD patients (P = 0.9613). However, rs57095329 showed a significant association with the cognitive deterioration of the AD patients (p = 0.03598), the AA genotype of rs57095329 was observed with a trend of accelerating the cognitive deterioration in AD patients (Figure 1 B), indicating that the AA genotype of rs57095329 in miR146a was a risk factor for cognitive decline in AD.

### 3.4 The influence of the two functional SNPs on mature miR146a expression

**Figure 2:**
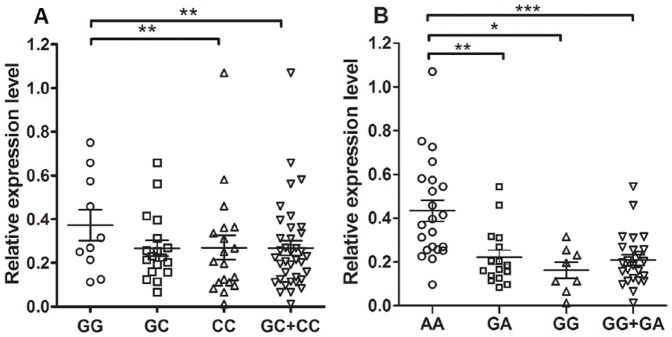
miR146a expression level with different genotypes of rs2910164 (A) and rs57095329 (B) in healthy individuals. The mature miR146a expression in PBMCS was analyzed in healthy individuals. The horizontal line indicates the mean expression level with each genotype groups. *p<0.05; **p<0.01; ***p<0.001.

Although the AD groups who have certain disease were excluded in advance, The AD samples is still difficult to meet the requirements of these functional study because some subjects were also suffered from other diseases in addition to AD that cannot be ruled out and some of them also receive the different drug treatment. These factors for each AD patients may influence gene expression, in turn effect the accuracy of this experiment. Therefore, to explore the influence of the SNPs on the expression of mature miR146a, we examined the expression of the different genotypes of miR146a in PBMCs that were obtained from the healthy subjects with more homogeneous background. The results are shown in Figure 2. The mean level of miR146a from subjects with the GG genotype of rs2910164 was significantly higher than those with the GC+CC genotypes (p = 0.0018) (Figure 2A). Individuals with the AA genotype for rs57095329 had significantly higher levels of miR146a than the GA+GG genotypes (p = 0.0002) (Figure 2B). The data indicated that the two SNPs are both functional and could affect the expression of miR146a. Notably, the mature miR146a level was increased 2.08-fold in individuals with the AA genotype compared to those with the GG+GA genotypes for rs57095329, which is higher than the 1.42-fold increase found in the GG genotype compared to the CC+GC genotypes of rs57095329, suggesting that the rs57095329 SNP has a stronger influence on the levels of mature miR146a.

### 3.5 The influence of miR146a polymorphisms on the production of IL-6 and IL-1β

**Figure 3:**
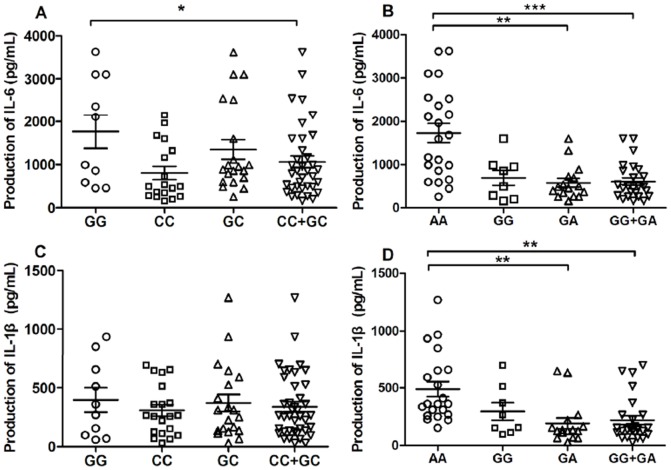
The production of IL-6 and IL-1β with different genotypes of miR146a polymorphisms in PBMCs under the LPS stimulation. (A) The IL-6 production with rs2910164 polymorphism; (B) The IL-6 production with rs57095329 polymorphism; (C) The IL-1β production with rs2910164 polymorphism; (D) The IL-1β production with rs57095329 polymorphism. The horizontal line indicates the mean expression level with each genotype groups. *p<0.05; **p<0.01; ***p<0.001.

We further examined the expression of the proinflammatory cytokines IL-6 and IL-1β in the PBMCs from individuals with different genotypes after stimulation with LPS. The results showed that the GG genotype of rs2910164 exhibited higher IL-6 expression than the CC+GC genotypes (p = 0.0468), and the AA genotype of rs57095329 had significantly higher level of IL-6 and IL-1β than the GA+GG genotypes (p = 0.0249 and p = 0.0009, respectively) (Figure 3). Moreover, the AA genotype of rs57095329 showed a higher mean production of IL-6 and IL-1β than the GG genotype of rs2910164 (IL-6: 1789.48±1034.45 pg/ml versus 1462.67±1210.27 pg/ml; IL-1β: 497.20±304.63 pg/ml versus 396.70±327.29 pg/ml), indicating that the AA genotype of rs57095329 may more strongly stimulated the production of IL-6 and IL-1β.

## Discussion

SNPs present on precursor and mature miRNAs have been shown to influence the level of the mature miRNA and have been shown to be associated with various diseases [22], [23]. In the present study, two SNPs of functional significance in miR146a, rs2910164 and rs57095329, were chosen to evaluate their association with AD. As expected, our results confirmed that the two SNPs could both affect the levels of mature miR146a (Figure 2), which is consistent with previous reports [15], [17]. Moreover, our study identified that the AA genotype of the rs57095329 polymorphism, but not rs2910164, was associated with an increased risk for cognitive decline in AD patients. To our knowledge, this is the first study to report the potential role of genetic variants of miR146a in AD susceptibility.

The G/A polymorphism of rs57095329 is located in the promoter region of miR146a with a binding site for the V-Ets oncogene homologue 1 (Ets-1). The A allele of rs57095329 interferes with Ets-1 binding and leading to a higher expression level of mature miR146a [17] (Figure S1). Meanwhile, increasing evidence indicates that miR146a plays a role in stimulating the inflammation response in the brains of AD patients [12]–[14], and our results also confirm that the AA genotype could increased the mature level of miR146a and elevated the level of proinflammatory cytokines IL-1β and IL-6, so we speculate that the change in the expression level of miR146a by the rs57095329 polymorphism may in turn regulated the inflammation response in the brains of AD patients, which could further contribute to the AD susceptibility. Notably, Ets-1 was reported to be widely expressed in the cortex and hippocampus, and particularly high in the brains of AD patients [24], suggesting that rs57095329 may have a stronger effect on the expression of miR146a on account of the high level of ETS-1 in AD brain, also supported this view.

ApoE polymorphic alleles are the main genetic determinants of LOAD risk [4]. Our studies showed that although the rs57095329 polymorphism was associated with AD risk, it did not differ across the ApoE4 genotype groups, demonstrating no significantly effect no significant relationship with the ApoE gene on the AD susceptibility. Numerous findings indicate that ApoEε4 has potential proinflammatory functions or reduced anti-inflammatory activity in AD [27]–[31]. Our study showed that the AA genotype of rs57095329 could increase the expression level of miR146a and was positively correlated with the expression of inflammatory cytokines, also presenting a stimulatory effect on the inflammatory response. However, no significantly synergistic effect was found between the miR146a polymorphism and the APOE ε4 allele in this case-control study. Nevertheless, due to the relatively small number of ApoE subgroups and the less clear relationships between the ApoE allele, miR146a and neuroinflammation in AD, whether there is a correlation between miR146a and ApoE in the pathology of AD will require further study. In another association analysis of the EOAD/LOAD subgroups, our results showed that the AA genotype was associated with an increased risk of AD in the EOAD subgroups. It is well known that mutations in the APP, PS1 and PS2 genes have been mainly discovered in patients with EOAD [3]. APP encodes the amyloid precursor protein found in plaques, and PS1 and PS2 are fundamental components of the γ-secretase complex involved in the cleavage of APP. All three of these genes contribute to the production of Aβ [1]. As mentioned previously, miR146a may play a role in the production of Aβ through the miR146a-TSPAN12-ADAM10 (α-secretase) pathway [25], [26] (Figure S1). We hypothesized that in addition to the regulating role of miR146a polymorphisms on the neuroinflammation, rs57095329 may also influence the metabolism of APP and eventually increase the generation of Aβ42, thus contributing to the risk of EOAD.

Numerous reports have associated neuroinflammation with cognitive decline in AD [27], [28], [29], [30]. A clinical study recently reported that AD individuals with a high inflammatory score had a more accelerated decline in their MMSE score over a 3-year period than those with a low inflammatory score [31]. The elevated concentrations of pro-inflammatory cytokines, such as IL-1β and IL-6, were also associated with impaired cognitive performance [32], [33]. Notably, as the positive regulator of inflammation in AD, the elevated levels of miR146a may increase the expression of pro-inflammatory cytokines in AD patients. Therefore, it is possible that the functional polymorphism in miR146a may contribute to the cognitive degeneration in AD patients by influencing the inflammatory process in the brain. Our data showed a positive association between the risk-associated AA genotype of rs57095329 and the cognitive deterioration of AD patients, in agreement with the phenomenon that higher levels of IL-1β and IL-6 were produced individuals with the AA genotype as a result of the stimulation. Although the mechanisms underlying this association cannot be directly inferred from this study, we believe that AD patients with the rs57095329 AA genotype may produce a higher amount of miR146a, which enhances the inflammatory response and leads to the progression of AD. However, due to the limited number of AD samples in our study, this association should be further verified in a larger cohort.

The frequency of the two SNPs of miR146a in a different healthy population was also determined. We sought to collect data from a large representative sample and found that the rs2910164 allele frequency is highly heterogeneous among the different populations. Among Asian populations, the C allele frequencies varied from 32.5–63.8% [17], [18], [34], [35], [36], [37], [38], [39]. In Caucasians, the C allele frequencies varied from 20.00–52.00% in German, Italian, Finnish, Turkish and American populations [40], [41], [42], [43], [44], [45], and it was lower than in the Asian populations in general. The Turkish population showed the lowest mutant allele frequency (20.0%) [43]. Notably, even in the Chinese populations from different regions, the frequency of rs2910164 still varied widely [17], [34], [41], [46], [47], [48], [49]. For instance, compared to the data from the northern Chinese population (Chinese Han in Beijing, CHB) using HapMap data (http://snp.cshl.org/cgi-perl/gbrowse/hapmap27_B36/), the frequency of the C alleles in the southern Chinese population in our study was higher (62.5% versus. 44.4%), indicating a different distribution pattern of miR146a rs2910164 in China. Thus, our study does not rule out the possibility that the rs2910164 polymorphism may indicate susceptibility to AD in other ethnic groups. The frequency of the G allele of rs57095329 among the healthy controls in the southern Chinese population was 17% in our study, which was similar to the frequencies observed in a healthy southern Chinese population and slightly lower than those previously observed in a Thai population (23%) [15] (Table S2). However, to date, the frequencies of the rs57095329 alleles have only been reported by two studies in Thai and Chinese populations, and no other data have been reported for other populations or have been recorded in the HapMap database [15], [17]. Further study on the variability of the rs57095329 frequency in different ethnic populations is needed.

Some potential limitations of our study should be addressed. The results of the case-control analysis in the EOAD/LOAD or ApoE subgroups remain preliminary due to the small number of subjects, and further investigation of the miR146a polymorphisms with a larger and more ethnically diverse population of AD patients is warranted. Due to the inconvenience of obtaining a number of brain samples, we only detected the influence of the two SNPs on the expression of miR146a and the production of IL-6 and IL-1β in PBMCs obtained from the healthy cases, and we are aware that the measurement of miR146a expression in the peripheral blood may not fully reflect its expression in the central nervous system. In addition to the functional polymorphisms of miR146a, other functional polymorphisms of other genes in each individual may also influence the production of IL-1β and IL-6, and this is a factor that we cannot exclude.

In conclusion, our study identified a significant association between the rs57095329 polymorphism in the promoter of miR146a and the risk of AD for the first time. The AA genotype of the rs57095329 polymorphism was associated with an increased risk for cognitive decline in AD patients. Therefore, genetic variation in miR146a may play a role in the regulation of AD development. Our future studies will focus on the mechanisms underlying this connection, and the association between rs57095329 and AD should be further examined in a large sample and in different ethnic groups.

## Supporting Information

Figure S1 **Schematic illustration of how the two SNPs alter the expression of miR146a and influence the pathological process of AD.**
(DOC)

Table S1 **The frequencies of haplotypes of miR146a gene in patients and controls.**
(DOC)

Table S2 **Mutant allele frequency of miR146a rs57095329 A>G genetic polymorphism of healthy individuals in the reported groups.**
(DOC)
