# Supplementary material for: A Functional Polymorphism in the Promoter Region of MicroRNA-146a Is Associated with the Risk of Alzheimer Disease and the Rate of Cognitive Decline in Patients
Source: PLoS One. 2014 Feb 25;9(2):e89019. doi: 10.1371/journal.pone.0089019 (PMC3934871; doi:10.1371/journal.pone.0089019)
Supplement: Figure S1 — Schematic illustration of how the two SNPs alter the expression of miR146a and influence the pathological process of AD. (DOC) [file pone.0089019.s001.doc]

**
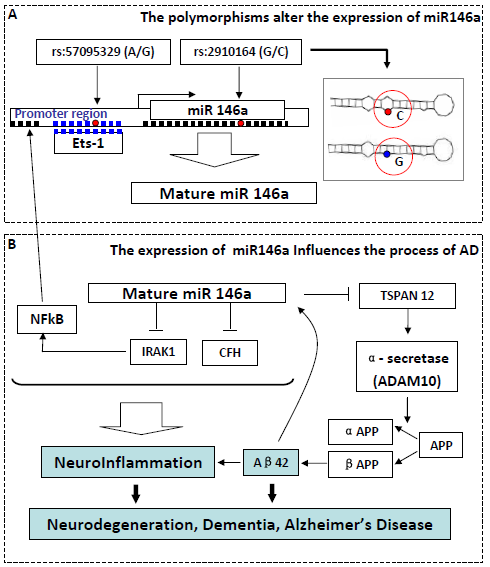
**

**Figure S1. Schematic illustration of how the two SNPs alter the expression of miR146a and influence the pathological process of AD.** (A) The rs2910164 polymorphism precursor results in a mismatch in the stem region and affects the expression of mature miR146a. Another polymorphism, rs57095329, is located in the promoter region of miR146a and has been reported to affect the availability and binding affinity of Ets-1, thus regulating the expression of miR146a. (B) The upregulated miR146a could negatively regulate CFH, a highly abundant oppressor of immune and inflammation in brain, thus promote the neuroinflammation in AD. Moreover, the expression of miR146a is also regulated by NFkB (a transcription factor for miR146a) and could negative regulate the IRAK1, plays a role in regulating the NFkB-IRAK1 signaling loop, ultimately accompanied with the activation of NFkB, thus showing the effect on increasing the inflammation in the neurodegenerative process of AD. Additionally, TSPAN12 is also a target of miR146a, which could exhibit positive regulation in the ADAM10-dependent processing of αAPP and may influence the generation of Aβ42 peptides, thus contributing to the neurodegenerative processes in AD.
