# Supplementary material for: A Functional Polymorphism in the Promoter Region of MicroRNA-146a Is Associated with the Risk of Alzheimer Disease and the Rate of Cognitive Decline in Patients
Source: PLoS One. 2014 Feb 25;9(2):e89019. doi: 10.1371/journal.pone.0089019 (PMC3934871; doi:10.1371/journal.pone.0089019)
Supplement: Table S1 — The frequencies of haplotypes of miR146a gene in patients and controls. (DOC) [file pone.0089019.s002.doc]

**Table S1. The frequencies of haplotypes of miR146a gene in patients and controls**

| **rs2910164–rs57095329** | **Case n(%)** | **Control n(%)** | **OR (95% CI)** | **P-value** |
| --- | --- | --- | --- | --- |
| **C-A** | 366(62.67) | 353(58.83) | 1.0000 (reference) | **-** |
| **G-G** | 52(8.90) | 80(13.33) | 0.6815 (0.36-1.05) | 0.0956 |
| **G-A** | 152(26.03) | 145(24.17) | 0.9322 (0.76-1.35) | 0.2331 |
| **C-G** | 14(2.40) | 22(3.67) | 0.7219 (0.53-1.14 ) | 0.1763 |
