# Supplementary material for: A Functional Polymorphism in the Promoter Region of MicroRNA-146a Is Associated with the Risk of Alzheimer Disease and the Rate of Cognitive Decline in Patients
Source: PLoS One. 2014 Feb 25;9(2):e89019. doi: 10.1371/journal.pone.0089019 (PMC3934871; doi:10.1371/journal.pone.0089019)
Supplement: Table S2 — Mutant allele frequency of miR146a rs57095329 A>G genetic polymorphism of healthy individuals in the reported groups. (DOC) [file pone.0089019.s003.doc]

**Table S2. Mutant allele frequency of miR146a rs57095329 A＞G genetic polymorphism of healthy individuals in the reported groups**

| **Country (City)** | **Number of Alleles** | **Frequency (G)** | **References** |
| --- | --- | --- | --- |
| China (HongKong) | 2304 | 0.20 | [15] |
| China (Shanghai) | 2160 | 0.16 | [15] |
| China(Chongqing and Guangzhou) | 1618 | 0.18 | [17] |
| Thailand (Bangkok) | 1964 | 0.23 | [15] |
| China (Shenzhen) | 600 | 0.17 | Present study |
